# Muscle anisotropy influences the phrenic nerve activation threshold in non-invasive electrical stimulation

**DOI:** 10.1007/s11517-026-03584-2

**Published:** 2026-05-20

**Authors:** Laureen Wegert, Marek Ziolkowski, Alexander Hunold, Tim Kalla, Irene Lange, Jens Haueisen

**Affiliations:** 1 Institute of Biomedical Engineering and Informatics, TU Ilmenau, Ilmenau, Germany; 2 neuroConn GmbH, Ilmenau, Germany

**Keywords:** Transcutaneuous electrical nerve stimulation, Artificial respiration, Multiscale modeling, Finite element method, Anisotropic conductivity

## Abstract

**Objective:**

Electric phrenic nerve stimulation is employed as a method of artificial ventilation, and computational models are utilized to assist in parameter selection. The majority of models assume isotropic tissue conductivity, although muscle tissue exhibits anisotropic properties. We aim to investigate the influence of anisotropic muscle conductivity on the results of phrenic nerve activation.

**Methods:**

To calculate the potential distribution, we used an anatomically detailed multi-scale model for non-invasive electrical stimulation in the neck, incorporating realistic muscle fiber orientations. Phrenic nerve activation thresholds were calculated using the McIntyre-Richardson-Grill nerve model. Anisotropy ratios ranging from 1:1 to 1:15 (transversal:longitudinal conductivities) were analyzed at constant corresponding isotropic conductivity. Additional simulations assessed the influence of muscle volume and electrode placement and quantified possible co-activation of other nerves in the neck.

**Main results:**

Increasing anisotropy ratios resulted in consistently higher phrenic nerve activation thresholds across all axon diameters (up to + 90%). Larger muscle volumes and electrode positions directly over a muscle further elevated the anisotropy effects. Considering anisotropic muscle conductivity increases the number of co-activated nerves.

**Conclusion:**

High-resolution models incorporating anisotropic conductivity are recommended for research studies on phrenic nerve stimulation.

**Graphical abstract:**

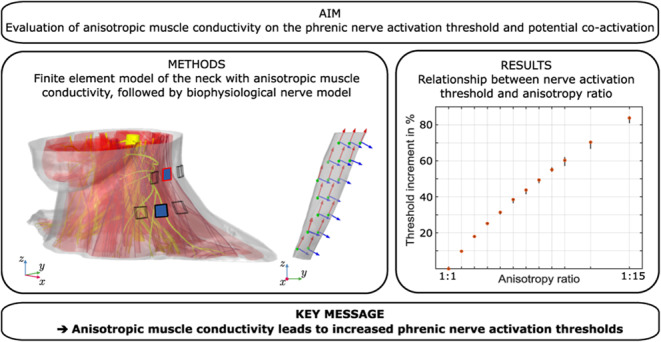

**Supplementary Information:**

The online version contains supplementary material available at 10.1007/s11517-026-03584-2.

## Introduction

Mechanical ventilation is a life-saving therapy frequently employed in intensive care units [1]. However, the inspiratory muscles are not actively utilized, which can result in ventilator-induced diaphragmatic dysfunction as an adverse effect [2–4]. The stimulation of the diaphragm innervating nerve (phrenic nerve) has been demonstrated to maintain the muscle activity and facilitate physiological breathing movements [5–7]. Invasive stimulation has been used successfully as a diaphragm pacemaker for tetraplegia patients [8]. However, this approach requires surgical implantation of electrodes and is not well-suited for temporary intervention. Consequently, semi-invasive techniques have been proposed, such as percutaneous or catheter-based electrode placement in close proximity to the phrenic nerve (e. g., with intravenous leads) [9–11]. Additionally, non-invasive electric phrenic nerve stimulation strategies have been developed [12–14]. To achieve efficient stimulation while maintaining patient comfort and avoiding off-target activation, a wide range of stimulation parameters is available and must be selected carefully. The implementation of computational models and electromagnetic simulations can facilitate the selection of appropriate stimulation parameters. These models predict nerve activation thresholds and guide the design of optimized stimulation protocols for efficient and safe stimulation. Therefore, the development of detailed and accurate models, as well as the systematic characterization of tissue properties, is imperative [15, 16]. First models for non-invasive electric [17], non-invasive magnetic [18], and percutaneous [19] phrenic nerve stimulation were presented and reviewed. The non-invasive methods aim to activate the phrenic nerve in the neck region.

The anatomical structure of the human neck is characterized by a high degree of complexity, comprising a variety of tissue types and organs, including muscles of diverse sizes and shapes. The characteristic fiber structure of muscle tissue leads to anisotropic tissue properties, such as its electric conductivity. The influence of anisotropic conductivity behavior on the electric potential and electric field distributions is known for various biomedical applications. For instance, white matter anisotropy has been demonstrated to influence the electric field distributions in electroencephalography [20–22] and transcranial electric stimulation [23]. Similarly, muscle anisotropy has been shown to affect the simulation results in electromyography [24, 25], and heart muscle anisotropy in electrocardiography [26, 27]. The anisotropic properties of the surrounding tissues also influence the activation thresholds in peripheral nerve stimulation and the recruitment patterns [28, 29]. Similarly, anisotropy in central white matter tracts shapes current flow and neural activation during brain stimulation [30, 31]. In summary, the previous work demonstrated that anisotropic conductivity exerts a qualitative and quantitative influence on simulation results. This emphasized the importance of considering anisotropic tissue behavior.

For skeletal muscle tissue, research reviews conducted by Baumgartner et al. [32] and Gabriel et al. [33] yielded divergent values for the conductivity in the longitudinal and transversal fiber direction. This results in a variety of anisotropy ratios from close to 1 (isotropic) [34] up to 15 [35]. Furthermore, the measurement frequency during electrical bioimpedance spectroscopy has been demonstrated to be a contributing factor. An increased measurement frequency yields a decreasing effect on the anisotropy ratio [34].

However, only a few electromagnetic models in the field of peripheral nerve stimulation included anisotropic muscle conductivity behavior. Moreover, these models are based on geometric approximations regarding the shape of the muscles or the fiber direction [36, 37]. In order to perform a comprehensive evaluation of phrenic nerve stimulation in the neck region, it is necessary to employ a detailed model that realistically incorporates anisotropic muscle conductivity, muscle geometry, and the direction of muscle fibers.

In this study, we aim to systematically evaluate how anisotropic electrical conductivity in the cervical muscles affects the phrenic nerve activation threshold using an anatomically detailed neck model. We specifically investigate whether incorporating muscle anisotropy is necessary for accurate prediction or whether isotropic assumptions are sufficient. In order to address the variability reported in the literature, different anisotropy ratios are analyzed. In addition, we examine the interaction between muscle anisotropy and stimulation electrode position, as well as variations in muscle volume, to reflect anatomical differences. By systematically varying these parameters in a controlled computational modeling framework, we aim to quantify their relative impact on phrenic nerve activation thresholds. The study provides a basis for the development of individualized models and clarifies whether advanced imaging techniques, such as diffusion tensor imaging (DTI), are required for accurate modeling.

## Methods

### Finite element model of the neck and the phrenic nerve

**Fig. 1:**
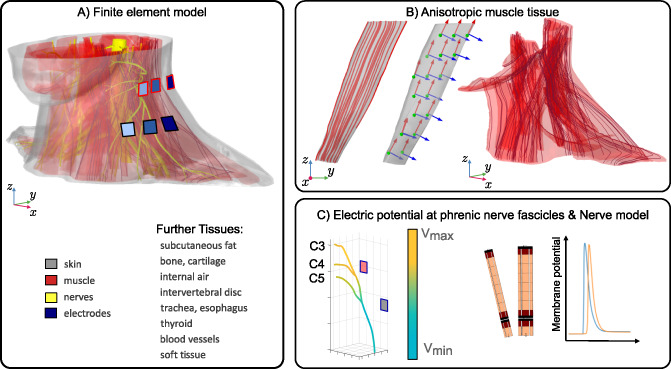
Model overview. **A**) Finite element model of the neck with highlighted nerves and muscles. Three electrode pairs (anode red, cathode black surrounding) are marked by blue squares with different shading. **B**) Anisotropic muscle fibers for the sternocleidomastoid muscle with stream-lines and directions of the local basis system (red, blue and green arrows showing the basis vectors) from flow simulation. **C**) Scalar electric potential mapped to the three fascicles of the phrenic nerve and used for the biophysiological nerve model (multicompartment model) analyzing the membrane potential and triggering of action potentials

To calculate the nerve activation threshold of the phrenic nerve, the multi-scale model of the neck and the phrenic nerve published in Wegert et al. [17, 38] was used and developed further. The macro-scale finite element model exhibited a high degree of anatomical detail, providing a comprehensive representation of the cervical region. The neck model was bounded by cut planes at the height of vertebrae C1 and T1. The neck model was built modular, incorporating specific anatomical components such as nerves and muscles, which were provided by the *BodyParts3D* platform [39]. In total, 13 tissue types were included in the neck model: skin, subcutaneous fat, muscle, bone, cartilage, nerves, thyroid, blood vessels, internal air, intervertebral discs, esophagus, trachea, and soft tissue (Fig. A.1). Two surface electrodes with a size of 1 cm × 1 cm were placed at the skin, in vicinity to the posterior border of the sternocleidomastoid muscle, with a center-to-center distance of 3*.*5 cm. The cathode was placed at the height of the cricoid cartilage. Two additional stimulation electrode pairs were added by shifting the electrodes 2 cm in the anterior and posterior directions. The electrodes’ shifted position resulted in locations superficial to the sternocleidomastoid and trapezius muscle, as depicted in Fig. 1. In all three electrode configurations, the upper electrode was used as anode (red surrounding), and the bottom electrode as cathode (black surrounding). An additional ground electrode of the same size was positioned at the back of the neck.

The modular structure of the neck model allows adaptation to individual patient anatomies. For clinical translation, patient-specific scaling can be performed at different levels of complexity, e.g. based on segmented magnetic resonance imaging (MRI) data or ultrasound measurements. The present study was designed as a controlled a priori investigation to isolate the influence of muscle conductivity anisotropy. To ensure controlled parameter analysis, geometric parameters such as neck circumference and subcutaneous fat thickness were kept constant, as changes in electrode-nerve distance would have introduced a dominant confounding effect. In order to represent the anatomical variability of muscle tissue in the population, five volume ratios of individual muscles were created, resulting in five macro-scale neck models. Due to the modular model structure, it was possible to scale only the muscle compartments. Therefore, the muscles were scaled in their cut surface area and consequently in their volume. The volume ratios were 75 %, 90 %, 110 %, and 125 % based on the variability of the sternocleidomastoid muscle in Migotto et al. [40]. The scaling process was done with open-source tools from MeshLab [41]. An increase in muscle volume was associated with a decrease in the volume of the surrounding soft tissue, and vice versa.

We integrated the previously used meso-scale finite element model of the phrenic nerve with the three fascicles to the nerve rootlets C3, C4, and C5, and anisotropic nerve conductivity [17] into each macro-scale neck model described above. The combined model is shown in Fig. 1A and was built with COMSOL Multiphysics (COMSOL AB, Stockholm, Sweden, Version 6.0.405). The tetrahedral mesh of the combined macro- and meso-scale model consisted of approximately 95 million elements.

The scalar electric potential distribution was computed under the quasi-stationary approximation, which is valid for low-frequency electrical stimulation where wave propagation and displacement currents can be neglected [42, 43]. In COMSOL Multiphysics, this was implemented using the *Electric Currents* physics interface and a stationary solver, which provides the solution of the quasi-stationary field for a given stimulation amplitude. The following boundary conditions were incorporated (Fig. A.1). The lateral surface of the neck with skin tissue served as an electric insulator with *J*_*n*_ = 0. The electric current was injected at the surface of one pair of the stimulation electrodes with normal current density (*J*_*n*_) of 20 mA*/*cm^2^. The three pairs of electrodes were evaluated separately. The ground potential *V* = 0 was defined at the additional electrode located at the back of the neck model. Floating potentials were assigned at the cut planes to ensure possible current flow in the head and the thorax.

First, isotropic material properties were assigned to the different tissue types based on Wegert et al. [17] and Baumgartner et al. [32]. Stimulation pulses with pulse widths of 10 *µ*s *−* 1 ms are commonly used in phrenic nerve stimulation [7, 12]. Due to the short pulse widths and finite rise times, the spectral content of such pulses extends from low frequencies up to approximately 100 kHz. Since tissue conductivity exhibits frequency dependence in this range, conductivity values within this band were considered. For the quasi-static volume conductor simulations, a representative mean conductivity value across this frequency range was used. The values are summarized in the Supplementary Material (Fig. A.1).

Some tissues from *BodyParts3D* had overlapping parts in the geometry, e. g. blood vessels or nerves with surrounding muscles. Duplicate areas in the tissues were addressed by the order of material allocation. The material properties assigned last for each component corresponded to the tissues for the calculation.

The calculation of the electric potential was performed using second-order tetrahedral elements, yielding approximately 127 million degrees of freedom. The potential values located at the midline of the phrenic nerve fascicles were exported and subsequently utilized as extracellular potentials for the micro-scale nerve model, which was based on the McIntyre-Richardson-Grill (MRG) model for myelinated axons [44]. With the micro-scale model, we calculated the nerve activation threshold for phrenic nerve fibers by evaluating the membrane potential. Therefore, a sample stimulation pulse was employed, which modulates the potential distribution. The potential distribution was iteratively scaled until action potentials were detected within the time course of the membrane potential, which was associated with the activation of the nerve fibers (Fig. 1C). The calculation was done in the *NEURON* simulation environment with Python [45]. An overview of the calculation of the nerve activation threshold is given in the Supplementary Material (Fig. A.2).

### Anisotropic muscle conductivity

From the literature, it is known that muscle tissue exhibits different conductivity values in the longitudinal *σ*_long_ and the transversal *σ*_trans_ fiber directions. These values were derived from the equivalent isotropic electrical conductivity, denoted as *σ*_iso_, employing the approach presented in Güllmar et al. [21] and Wolters [46]. This approach represented the anisotropic conductivity tensor as an ellipsoid, characterized by a constant volume and the semi-axes *a* = *σ*_long_ and *b* = *c* = *σ*_trans_:

1 $$\frac{4}{3}\pi {\sigma }_{iso}^{3}=\frac{4}{3}\pi {\sigma }_{long}{\sigma }_{trans}^{2}$$

The ratio *r* between the transversal and longitudinal conductivity was introduced as follows:

2 $$r =\frac{{\sigma }_{trans}}{{\sigma }_{long}}$$

The longitudinal and transversal conductivities were calculated from the isotropic value by transforming Equation 1 and Equation 2 as follows:

3 $${\sigma }_{long}={\sigma }_{iso} {r}^{-2/3}$$

4 $${\sigma }_{trans}={\sigma }_{iso} {r}^{1/3}$$

Reported anisotropy ratios [32, 33] range from close to 1:1 [34] up to 1:15 [35]. Here, we investigated ratios ranging from 1:1 (isotropic) up to 1:10, with step size 1 incremental increases in *r*, along with 1:12, and 1:15. For the corresponding isotropic conductivity, a value of 0*.*2986 S*/*m was utilized as presented in our previous study [17]. The resulting longitudinal and transversal muscle conductivities are summarized in Table 1.

**Table 1:** Anisotropic conductivity values in longitudinal and transversal direction for different anisotropy ratios *r* and the corresponding isotropic conductivity *σ*_iso_ of 0*.*2986 S*/*m

		Anisotropic ratio *r*					
		1:1	1:2	$\cdots$	1:10	1:12	1:15
$\sigma_{\mathrm{long}}$	in S*/*m	0.2986	0.4740	$\cdots$	1.3860	1.5651	1.8161
$\sigma_{\mathrm{trans}}$	in S*/*m	0.2986	0.2370	$\cdots$	0.1386	0.1304	0.1211

The muscle compartments that were located within 20 mm of the phrenic nerve and the electrodes were modeled with anisotropic conductivity. These included the sternocleidomastoid, splenius, scalenus, levator scapulae, longissimus, longus, iliocostal, omohyoid, semispinalis, platysma, longus colli, obliquus capitis inferior, trapezius (descending part), sternohyoid, sternothyroid, palatopharyngeus, stylopharyngeus, pharyngeal constrictor, and rectus capitis posterior major muscles on the left body side (stimulation side). These muscle compartments were assigned with homogenized anisotropic electrical conductivity described by the following diagonal conductivity tensor:

5 $$\underline{\underline{\sigma}}=\left[\begin{array}{ccc}{\sigma }_{long}& 0& 0\\ 0& {\sigma }_{trans}& 0\\ 0& 0& {\sigma }_{trans}\end{array}\right]$$

where *σ*_long_ and *σ*_trans_ denote electrical conductivities in directions longitudinal and transversal to the muscle fiber axis, respectively.

For the remaining muscles, we applied the isotropic muscle conductivity value.

This includes, for example, the muscle compartments on the right side of the neck. In order to assign the longitudinal and transversal conductivity values of the conductivity tensor with respect to the fiber direction, it was necessary to calculate local coordinate systems. In other studies, information about the fiber direction could be taken from diffusion tensor images, but this was not available for our model. Consequently, an alternative approach was devised, employing the *Curvilinear Coordinate* interface from COMSOL Multiphysics to consider the fiber direction. Therefore, an additional flow-like simulation was conducted (Fig. A.1), with the definition of an inlet and outlet. The calculated streamlines were comparable to the direction of muscle fibers. The curvilinear coordinate system for each muscle was defined by a system of local base vectors *{***e**_1_*,* **e**_2_*,* **e**_3_*}*. The curvilinear coordinate system was adapted to the shape and the geometry of the muscle, with *{***e**_1_*}* (Fig. 1B, red) following the direction of the muscle fibers. The inlet node was defined at the proximal end of each muscle bundle, and the outlet node was at the distal end of the muscle. To visualize the curvilinear system’s orientation, streamlines of the internal muscle vector field were generated with a consistent density throughout each muscle’s cross-section. Figure 1B presents a magnified view of the streamlines and local base vectors within the anisotropic muscle compartments for the sternocleidomastoid muscle.

### Evaluation parameters

The activation of the phrenic nerve was represented by the nerve activation threshold (Fig. A.2). The parameter was defined as the minimum current required to trigger an action potential with a standardized reference pulse (cathodic, monophasic, pulse width 150 µs). For long-term stimulation, symmetric biphasic charge balanced pulses are commonly used but lead to increased thresholds. Alternative biphasic stimulation strategies, such as asymmetric waveforms or introducing an interphase delay, have been shown to substantially reduce threshold differences [17]. These strategies can approach monophasic activation thresholds while maintaining charge balance. Therefore, we used the monophasic pulse as reference signal. We evaluated the nerve activation threshold for axons with different diameters for the three fascicles separately with an accuracy of 1 mA. The mean value over the fascicles was calculated. The diameter of the modeled axons ranged from 7 to 14 µm, a range that corresponds to the majority of phrenic nerve axons [47, 48]. The nerve activation thresholds of the phrenic nerve fibers were calculated for the anisotropy ratios ranging from 1:1 (isotropic) to 1:15 for the standard electrode position, which corresponded to the middle pair of electrodes. We investigated the absolute and relative differences of the nerve activation thresholds resulting from anisotropic muscle conductivity values. The relative difference was evaluated by calculating the median difference and the quartiles over the evaluated fiber diameters.

To evaluate co-activation, the MRG-model was applied to the main segments of the following peripheral nerves: the brachial plexus (three segments), the ansa cervicalis, the great auricular nerve, the long thoracic nerve, the vagus nerve, the subclavian nerve, the supraclavicular nerve (two segments), and the transverse cervical nerve (two segments). For each anisotropy ratio, the stimulation current required to elicit an action potential in all fascicles of the phrenic nerve was determined. This current was intentionally used as the reference operating point. Selective stimulation is defined as activation of the target nerve without recruitment of non-target nerves at the same stimulation amplitude. Therefore, co-activation was evaluated exclusively at this physiologically relevant stimulation intensity rather than across the entire recruitment curve. For each non-target nerve segment, it was assessed whether the applied current elicited an action potential (binary outcome: activated/not activated). Due to the lack of detailed fiber diameter distributions for the potentially co-activated nerves, three representative fiber diameters (12*.*8 µm, 10 µm, and 7*.*3 µm) were chosen. These diameters correspond to typical ranges of large motor, sensory, and smaller myelinated fibers as described in the classification by Erlanger et al. [49]. The selected diameters serve as representative values and do not imply that all fiber types are present in each modeled nerve. Selectivity was qualitatively analyzed based on the number of activated non-target nerve segments related to the total number of nerve segments [50, 51].

The evaluation of the phrenic nerve activation thresholds was repeated for the other two electrode positions and the varied muscle volumes. A comparison of the thresholds was conducted with the standard electrode position and the unscaled muscle volume.

##  Results

### Phrenic nerve activation and selectivity

First, the influence of anisotropic muscle conductivity values on the phrenic nerve activation threshold was evaluated for the standard electrode configuration and the unscaled muscle volume.

**Fig. 2:**
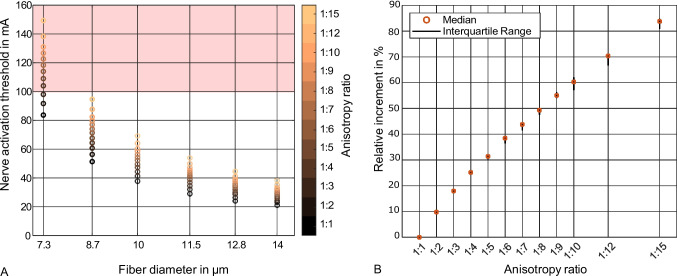
Phrenic nerve activation threshold for different fiber diameters and anisotropy ratios in absolute (A) and relative (B) values. A red background indicates remarkably high activation thresholds, as these values are not achievable in practical applications. Nerve activation thresholds increased with decreasing axon fiber diameter and increasing anisotropy ratio. Median values and interquartile ranges were calculated over the fiber diameters for the relative increment of the nerve activation threshold (B). Depending on the anisotropy ratio, the relative increment of the nerve activation threshold ranged from 9 to 83%, increasing with higher anisotropy ratios. The values obtained for the modeled fiber diameters were consistent, with an interquartile range of less than 5 %

The nerve activation threshold exhibited variations for the modeled fiber diameters and the anisotropy ratios (Fig. 2). The findings indicated that lower fiber diameters exhibited an elevated nerve activation threshold. A subsequent increase in anisotropy ratios led to higher nerve activation thresholds for all fiber diameters. The lowest nerve activation threshold was 21 mA, observed for a fiber diameter of 14 µm and an anisotropy ratio of 1:1. The highest nerve activation threshold was 149 mA, for a fiber diameter of 7*.*3 µm and an anisotropy ratio of 1:15. For the most prevalent fiber diameter of 10 µm, the nerve activation threshold exhibited a range from 37 mA (anisotropy ratio of 1:1) to 69 mA (anisotropy ratio of 1:15). For the largest fiber diameter of 14 µm, the nerve activation threshold differed from 21 mA (isotropic 1:1) up to 38 mA (anisotropy ratio of 1:15). For the smallest fiber diameter of 7*.*3 µm dimeter, the nerve activation threshold varied from 83 mA (isotropic 1:1) to 149 mA (anisotropy ratio of 1:15).

The absolute values of the nerve activation threshold were transferred to relative values of increment and plotted over the modeled anisotropy ratio (Fig. 2B). The application of an anisotropy ratio of 1:2 yielded a median increase of 9*.*7 % in phrenic nerve activation thresholds (relative to the results with the isotropic model), with an interquartile range of 0*.*18 %. For an anisotropy ratio of 1:15, the median relative increment was 83*.*8 % with 3*.*8 % interquartile range. The interquartile range exhibited a slight increase for higher anisotropy ratios, with a maximum of 4*.*9 % observed for the anisotropy ratio of 1:10. For each anisotropy ratio, the stimulation current required to activate phrenic nerve fibers with diameters of 12*.*8 µm, 10 µm, and 7*.*3 µm was subsequently applied to all modeled non-target nerves, and the occurrence of an action potential was evaluated. At the activation threshold of the 12*.*8 µm phrenic nerve fibers, fibers in several non-target nerves were also recruited (Fig. A.3). Activated segments included fibers of all diameters of the great auricular nerve, the supraclavicular nerve, the subclavian nerve (all segments), and the transverse cervical nerve (all segments). In addition, for large-diameter fibers, one segment of the brachial plexus, the ansa cervicalis, and the long thoracic nerve were activated. This resulted in 9 (12*.*8 µm), 7 (10 µm), and 6 (7*.*3 µm) activated off-target nerve segments (anisotropy ratio of 1:1). Higher anisotropy ratios led to an increased number of co-activated nerves and consequently reduced selectivity values across all evaluated non-target fiber diameters. For an anisotropy ratio of 1:15, the number of co-activated off-target nerves increased to 11 (12*.*8 µm), 8 (10 µm), and 8 (7*.*3 µm).

When the stimulation current corresponding to the activation of smaller phrenic nerve fibers (10 µm and 7*.*3 µm) was applied, additional non-target nerves were recruited across all non-target fiber diameters (Fig. A.4, Fig. A.5). These were e. g. further segments of the brachial plexus, the ansa cervicalis, and the long thoracic nerve. This further reduced selectivity, especially for off-target nerves with smaller diameter.

Overall, co-activation predominantly occurred in anatomically superficial nerves, such as the sensory transverse cervical nerve, the sensory supraclavicular nerve, and the sensory great auricular nerve, located close to the stimulation electrodes, whereas deeper nerve segments showed reduced recruitment.

### Altered electrode positions

**Fig. 3:**
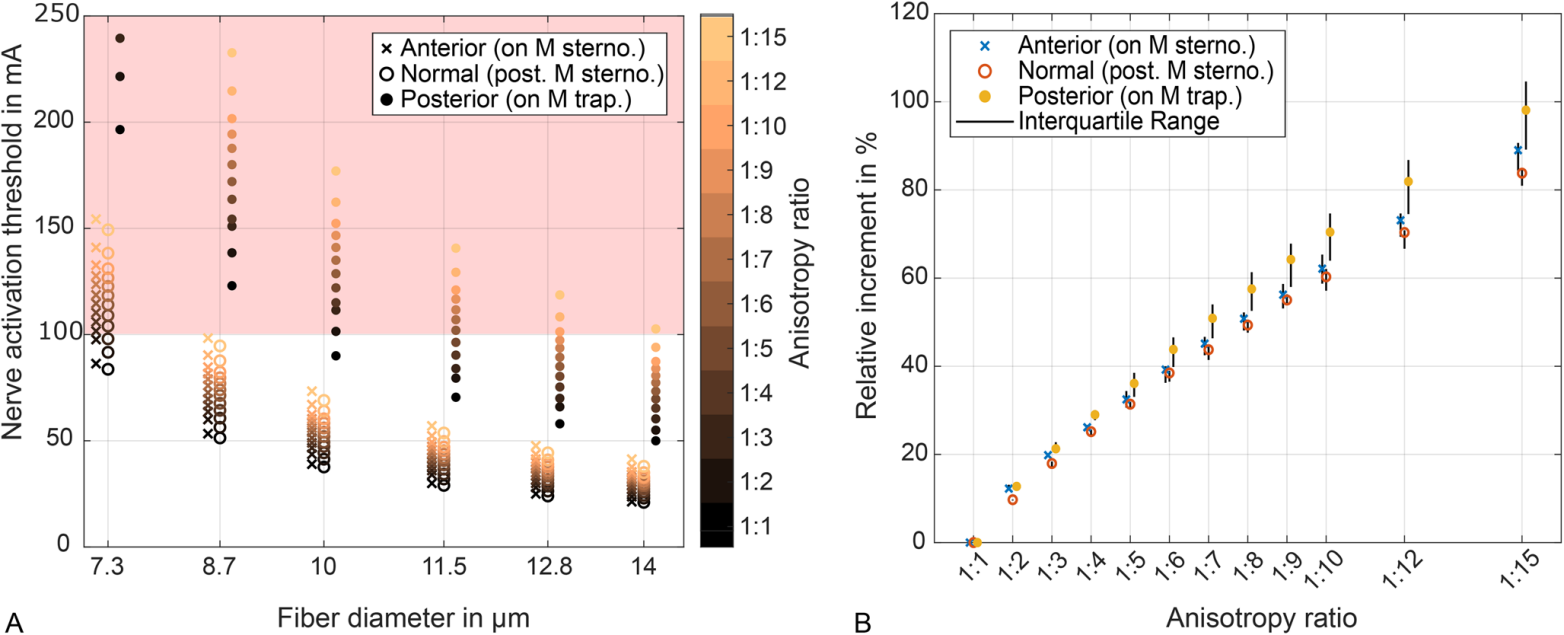
Nerve activation thresholds for the different electrode positions in absolute (A) and relative (B) values. A red background indicates remarkably high activation thresholds, as these values are not achievable in practical applications. The three electrode positions were marked with different symbols. Shifting the electrodes in anterior direction onto the sternocleidomastoid and in posterior direction onto the trapezius muscles increased the nerve activation thresholds for all fiber diameters. The highest values were observed for the electrodes on the trapezius muscle. A further increment in nerve activation threshold was observed with higher anisotropy ratios. The interquartile range increased with electrode placement superficial to the muscle and with a rising anisotropy ratio

Typically, the stimulation electrodes are placed at the posterior border of the sternocleidomastoid muscle. We varied this position in the anterior and posterior directions of 2 cm and evaluated how anisotropic muscle conductivity values further influence the phrenic nerve activation thresholds. The nerve activation thresholds were 38 mA, 39 mA, and 90 mA for the normal, anterior, and posterior electrode positions, respectively, for a fiber diameter of 10 µm and an anisotropy ratio of 1:1. Additional effects of the anisotropy ratio were observed and differed between the electrode positions. A higher anisotropy ratio resulted in an increased nerve activation threshold for all fiber diameters and electrode positions. For the 10 µm fiber diameter, the nerve activation threshold increased to 69 mA, 73 mA, and 177 mA for the three electrode positions. Figure 3A shows the nerve activation thresholds for the different electrode positions and all evaluated fiber diameters. Placing the electrodes in posterior direction (superficial to the trapezius muscle) highly increased the nerve activation threshold for the isotropic conductivity case (anisotropy ratio 1:1) by a factor of 2 *−* 3, depending on the fiber diameter. For placement in the anterior direction, the nerve activation increased slightly by about 1*−* 3 mA, depending on the fiber diameter. These differences were further increased with rising anisotropy ratios.

The increase in nerve activation threshold was transferred in relative values for the different anisotropy ratios (Fig. 3B). For the anterior position on the sternocleidomastoid muscle, the relative increase ranged from 12*.*3 % (interquartile range 1*.*2 %, anisotropy ratio 1:2) to 89 % (interquartile range 6*.*3 %, anisotropy ratio 1:15). For the posterior position on the trapezius muscle, the relative increase ranged from 12*.*7 % (interquartile range 0*.*2 %, anisotropy ratio 1:2) to 98*.*1 % (interquartile range 15*.*4 %, anisotropy ratio 1:15). Altered electrode positions onto the muscles showed a further increment of the nerve activation threshold up to 14*.*3 % due to anisotropic conductivity. This effect was most prominent for the posterior electrode position and anisotropy ratio of 1:15.

We observed an increased interquartile range for higher nerve activation thresholds. The maximum interquartile range was 15*.*4 % for the electrode position on the trapezius muscle.

### Variation of muscle volume

**Fig. 4:**
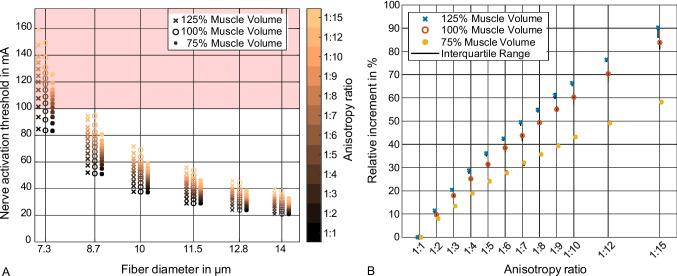
Nerve activation thresholds for three scaled muscle volumes in absolute (A) and relative (B) values. A red background indicates remarkably high activation thresholds, as these values are not achievable in practical applications. The varied muscle volumes were marked by distinct symbols. An increase in muscle volume was associated with an elevation in the nerve activation threshold for anisotropic conductivity, while a decrease in muscle volume was associated with a reduction in nerve activation threshold. For the isotropic case (anisotropy ratio 1:1), changes in the activation threshold were < 1 mA. Differences between the muscle volumes increased for higher anisotropy ratios

In a subsequent study, we investigated the impact of individual muscle volumes and anisotropic conductivity values on the phrenic nerve activation threshold. Figure 4A presents the nerve activation thresholds for the modeled fiber diameters and the scaled minimum and maximum muscle volumes. For an anisotropy ratio of 1:1 (isotropic), the nerve activation thresholds differed by less than 1 mA between the modeled volume ratios and all fiber diameters. Higher differences were observed for the implemented anisotropic conductivity values, and these differences increased with higher anisotropy ratios. An increase in muscle volume resulted in elevated nerve activation thresholds. For a sample fiber diameter of 10 µm, the nerve activation threshold increased from 37*.*6 mA (anisotropy ratio 1:1) to 72 mA, 70 mA, 69 mA, 64 mA, and 59 mA (anisotropy ratio 1:15) for the scaled muscle volumes of 125 %, 110 %, 100 %, 90 %, and 75 %, respectively.

Again, we converted the results into relative values, and we observed consistent values across all fiber diameters. The results from an anisotropy ratio of 1:15 corresponded to a relative increment of 90*.*3 %, 86*.*8 %, 83*.*8 %, 71*.*6 % and 58*.*2 % for the scaled muscles (decreasing volume order). The relative increment is shown in Fig. 4B. Differences were observed for the different muscle volumes. Higher muscle volumes resulted in higher increases of the nerve activation threshold for anisotropic conductivity across all anisotropy ratios. In accordance, a decrease in muscle volume resulted in a smaller increase in the median nerve activation threshold for rising anisotropy ratio. The differences between the minimum and maximum muscle volume increased with an increase in the anisotropy ratio.

##  Discussion

### Phrenic nerve activation and selectivity

While the anisotropic conductivity behavior of muscle tissue is well known, a wide range of anisotropy ratios has been published. In the presented study, we quantified the influence of varying anisotropy ratios within the neck during non-invasive electrical stimulation.

We employed an anatomically detailed finite element model with single muscle compartments. In each muscle compartment, anisotropic electrical conductivity values were incorporated through the introduction of local coordinate systems derived from flow simulations (Fig. 1B). In comparison to other FEM models [36, 52], our model enabled the separate consideration of single muscles with their specific fiber directions. We found that the fiber directions deviated from the global coordinate axis, particularly in the neck region. These deviations could be addressed by our approach.

As our study showed, muscle anisotropy influenced phrenic nerve activation thresholds (Fig. 2). Independent of fiber diameter, increasing anisotropy ratios led to elevated thresholds compared to the isotropic case. This observation is consistent with previous reports demonstrating altered recruitment behavior in anisotropic media [28]. The authors observed a larger increase in the activation thresholds of smaller fibers, resulting in higher currents being required to activate an equivalent number of fibers as in isotropic surrounding media.

**Fig. 5:**
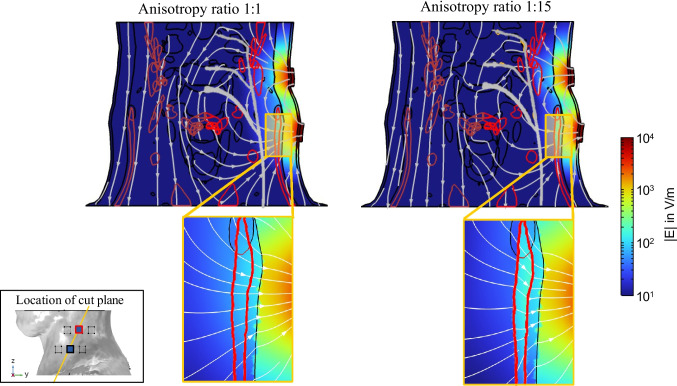
Electric field strength *|E|* distribution and normalized streamlines of current density within a sample cut plane through the stimulation electrodes for two sample anisotropy ratios. The position of the cut plane is shown in the lower left corner. Muscle compartments are highlighted with red outlines. The phrenic nerve is projected in the cut plane and highlighted in gray. A higher anisotropy ratio altered the current flow within muscle compartment regions

The observed effects can be attributed to the alterations in conductivity. For anisotropic conductivity values, the muscle compartments exhibited increased conductivity in the longitudinal fiber direction. The conductivity value was > 0*.*2986 S*/*m (Table 1) for anisotropy ratios from 1:2 to 1:15 compared to the surrounding soft tissue conductivity (0*.*3428 S*/*m). Consequently, the preferred direction of current flow was in the longitudinal direction of the muscle fibers. The superficial muscles, including the platysma, the sternocleidomastoid, and the trapezius muscle, led to a more superficial current flow. The direction of current flow and the alteration due to anisotropic conductivity are shown for a cut plane through the stimulation electrodes in Fig. 5. The normal current density was depicted as streamlines. The effects of anisotropic muscle conductivity became visible in the more irregular shape of the current streamlines.

From a clinical perspective, this implies that simplified isotropic tissue models may underestimate required stimulation amplitudes. When targeting deeper nerves such as the phrenic nerve, stimulation protocols may require adjustment of pulse amplitude or width to compensate for anisotropic effects when neglected in the model.

It was observed that some nerve activation thresholds reached amplitudes above 100 mA, which may exceed comfortable stimulation levels in transcutaneous nerve stimulation applications. However, it should be noted that these values were obtained with a pulse width of 150 µs. According to the strength-duration relationship, an increase in pulse width would result in a reduction of the nerve activation threshold [7, 12]. In addition, in a clinical context, the positioning of electrodes would be optimized on an individual basis to minimize the required current amplitudes. Consequently, the reported thresholds should be interpreted as theoretical upper bounds rather than clinically intended stimulation settings.

Co-activation was evaluated at the stimulation amplitude required for phrenic nerve activation. The analysis was intentionally restricted to a binary activation outcome (activated/not activated). The objective was to evaluate functional selectivity at the target activation threshold rather than to characterize full recruitment curves, which were not available as in the study of Dali et al. [51]. While this approach does not provide information about threshold current, it directly reflects whether off-target nerves would be recruited under clinically relevant stimulation conditions.

The results indicate that superficial nerve segments located close to the electrode configuration are most susceptible to co-activation. This finding provides a clear indication for further optimization of electrode geometry and stimulation parameters to improve selectivity. Based on established anatomical innervation patterns [53], co-activation of superficial cervical nerves (e. g., transverse cervical, supraclavicular, and great auricular nerves) would primarily result in localized sensory effects in the neck region. In contrast, activation of deeper structures such as the brachial plexus or the long thoracic nerve could induce motor responses in the upper limb or serratus anterior muscle. Within our simulations, activation thresholds of these superficial structures were lower than those of the phrenic nerve, indicating the need for optimization of the electrode configuration for im-proved selective stimulation. The vagus nerve represents a functionally critical structure due to its parasympathetic effects. However, the model predicts vagus nerve activation only at substantially higher current amplitudes compared to phrenic nerve activation, suggesting a comparatively low risk under selective stimulation conditions.

Compared to our previous work, in which co-activation was estimated using a surrogate electric field metric, the present analysis represents a methodological improvement. Activation is now determined by the biophysical MRG nerve model and provides a more physiologically interpretable assessment of selectivity.

###  Altered electrode positions

As shown in Fig. 3, modifying the electrode position in anterior and posterior direction resulted in increased nerve activation thresholds and an enhanced influence of anisotropic conductivity. In comparison to the reference electrode position, the altered electrode positions resulted in increased nerve activation thresholds of 12 % instead of 10 % (anisotropy ratio 1:2) and up to 98 % instead of 85 % (anisotropy ratio 1:15). As expected, increasing the distance between electrodes and the phrenic nerve increased thresholds in accordance with the literature [54].

The enhanced effect of anisotropy might be explained by the preferred current flow through tissues with the highest conductivity (Fig. 5). This was in the direction of the underlying muscle fibers of the sternocleidomastoid or trapezius muscle.

Clinically, this finding highlights that optimal electrode placement should consider not only geometric proximity to the nerve but also underlying muscle fiber orientation. Individual anatomical variability of the phrenic nerve [55] may further influence this interaction and should be considered in patient-specific stimulation planning.

### Variation of muscle volume

Individual differences in the muscle volume are known [40]. The variation can be attributed to gender differences, the individual’s muscle training status or pathologies such as muscle atrophy. To address this variance, the muscle compartments were scaled. Variations in muscle volume alone did not substantially alter activation thresholds in the isotropic case, which can be explained by comparable conductivity values of muscle and surrounding soft tissue (muscle 0*.*298 S*/*m, soft tissue 0*.*342 S*/*m). In the magnified view of Fig. 5 through the platysma muscle, a smooth transition of the electric field strength between the muscle and the surrounding soft tissue can be observed. In the anisotropic scenario, the electric field within the muscle is elevated relative to the surrounding soft tissue, which indicates the varying conductivities.

The scaled muscle compartments resulted in the formation of larger or smaller regions of anisotropic conductivity behavior. As discussed above, the current flow was found to be preferential in the longitudinal muscle fiber direction for the studied anisotropic cases. As a result, larger muscle volumes increased activation thresholds under anisotropic conditions (increase of 90 % instead of 85 % for an anisotropy ratio of 1:15). Conversely, reduced muscle volumes diminished this effect (increase of 58 % instead of 85 % for an anisotropy ratio of 1:15).

Clinically, this suggests that individual differences in the neck muscles influence stimulation efficiency when anisotropy is considered. Patients with smaller (superficial) muscle mass may require lower stimulation amplitudes, whereas pronounced musculature increases the required current. These findings support the need for individualized stimulation parameter adjustment.

### Comparison to anisotropic models of the nervous system

The results obtained in this study were consistent with those reported in other biomedical applications, such as the field of neuroscience. In electroencephalography, the effects of anisotropic conductivity were observed under the consideration of the fiber orientation from diffusion tensor images. Significant differences in the distribution of the electric field strength and the magnitude were shown, as well as effects on the inverse solution, as demonstrated by Güllmar et al. [21]. For transcranial current stimulation, the study by Shahid et al. [56] showed that anisotropic conductivity modulates the electric field strength in the brain with an effect that reaches up to 20 %. However, the topography remained similar. In addition, studies for deep brain stimulation have shown that modulation of electric field strength influences the activated area surrounding the stimulation electrode up to 18 % [57]. As demonstrated by Opitz et al. [58] for the application of transcranial magnetic stimulation, increments of up to 40 % of the maximum electric field strength in white matter regions were observed due to anisotropy. According to Fernandes et al. [59], the magnitude of the electric field strength within the spinal cord was affected by anisotropic conductivity for vertebral muscles during spinal cord stimulation. In the aforementioned applications, the utilization of detailed models and the consideration of anisotropic conductivity were recommended, particularly in the evaluation of specific activation regions. These outcomes align with our observations, wherein we noted increasing nerve activation thresholds with larger anisotropy ratios, which are comparable to decreased electric field strengths. High-resolution individual models taking into account anisotropic volume conduction are suitable for research purposes in electrical ventilation. In clinical applications, however, it might not be possible to construct such individual models for practical reasons.

###  Limitation

Due to the wide range of anisotropy ratios in the literature, we evaluated anisotropy ratios from 1:1 to 1:15, corresponding to the minimum and maximum values reported. We observed an increasing effect on the nerve activation thresholds with higher anisotropy ratios. Most literature on anisotropic muscle conductivity values reported an anisotropy ratio of approximately 1:3 [34, 36]. For the nerve activation threshold, this corresponded to an underestimation of 18 %. However, further measurements of muscle conductivity in the longitudinal and transversal directions are necessary to characterize these values, especially those of the neck muscles.

Since anisotropic conductivity primarily influences the region between the current injection position (the electrodes) and the evaluation position (the phrenic nerve), we incorporated anisotropic conductivity values in the regions near the electrodes and the phrenic nerve. One limitation of our study is that we did not consider the anisotropic conductivity values of the muscles on the non-stimulated side of the body.

Our volume conductor model was built based on the anatomical components of the platform *BodyParts3D* and represents the anatomy and the nerve course of a healthy volunteer. The data were derived from segmented MRI data. For clinical application, geometric personalization represents a critical step to account for a large range of neck circumferences. Patient-specific scaling can be performed at different levels of complexity. At a basic level, geometrical adaptation can be achieved using easily obtainable clinical parameters such as neck circumference, subcutaneous fat thickness, and superficial muscle thickness. These parameters may be derived from ultrasound measurements or caliper-based assessments. At a higher level of personalization, segmented MRI data may be used to derive patient-specific geometries. MR-DTI data could additionally be incorporated to define anisotropic conductivity tensors aligned with individual muscle fiber orientations. However, MR-DTI data of muscle tissue are not routinely available in clinical workflows and were not available for the model used here. In order to obtain information regarding the fiber orientation of muscle tissue, a flow simulation was conducted. The streamlines of the flow are consistent with the geometric muscle shape and were interpreted as fiber orientation. Variations may be observed at the beginning and end of the muscle. In the context of future individualized models, it is advisable to utilize DTI data for anisotropic conductivity directions. Muscle pennation was not modeled due to limited data for the cervical muscles. Instead, each muscle compartment was assigned a dominant fiber direction representing a homogenized anisotropic conductivity tensor. Individual differences in the phrenic nerve course and the number of fascicles are known [55], which may affect the phrenic nerve activation thresholds and the distinct influence of anisotropic conductivity.

After geometric adaptation, the individualized model can be used to optimize the stimulation protocols for improving selectivity and safety. Based on the calculated nerve activation thresholds, biphasic stimulation signals, and electrode configuration can be iteratively adjusted to achieve selective phrenic nerve activation while minimizing co-activation and avoiding small fiber and afferent activation. Personalizing stimulation protocols [60] include e.g. pulse width, waveform, stimulation frequency, and advanced stimulation paradigms such as temporal interference stimulation [61], which aims to limit superficial activation. Furthermore, multi-electrode arrays and current steering approaches [12, 62, 63] could be integrated to spatially focus the electric field toward the phrenic nerve while limiting superficial (e. g. transverse cervical nerve, supraclavicular nerve, great auricular nerve) or parasympathetic co-activation. These extensions indicate a rational subsequent phase in translating the presented modeling framework towards clinically optimized stimulation strategies.

In comparison to our previous publication [17], we integrated the detailed anatomy of the phrenic nerve (meso-scale) in the neck model (macro-scale). The built mesh within the phrenic nerve and the fascicles resulted in a coarser resolution. This limitation restricts the minimum fiber diameter that can be evaluated using the MRG nerve model, which was set at 7*.*3 µm. Further limitations result from the utilization of the MRG nerve model, such as the independent evaluation of nerve fibers with different diameters and their predefined properties for 7.3, 8.7, 10, 11.5, 12.8, and 14 µm. The nerve activation threshold for sample single pulses was evaluated. The utilization of diverse signal shapes, pulse widths, or pulse trains has been demonstrated to modify the nerve activation threshold [12, 17].

Additional limitations must be considered for the evaluation of the co-activation and the selectivity. Representative fiber diameters were used due to the lack of detailed histological data for all modeled nerves. Not all fiber types are present in every peripheral nerve, and the applied diameters should therefore be interpreted as sample values. Furthermore, all non-target nerves were weighted equally in the qualitative evaluation of the selectivity, independent of their functional role or fiber composition. Consequently, the selectivity evaluates anatomical co-activation rather than functional impact.

Additionally, co-activation of cervical muscles was not considered in the present simulations, as the primary objective was to evaluate nerve activation thresholds. However, muscle activation also influences perceived discomfort and functional selectivity during stimulation. Future extensions of the model may incorporate muscle fiber excitability to provide a more comprehensive assessment of stimulation-induced motor responses.

##  Conclusion

The present study was conducted to examine the effects of anisotropic muscle conductivity values within the neck on the phrenic nerve activation threshold. We provide insights into the current flow within the neck and the resulting effects on the phrenic nerve activation threshold and potentially co-activated nerves. We observed a high influence of anisotropic conductivity on the activation of the phrenic nerve, which was further influenced by the muscle size and the electrode position.

In order to minimize the occurrence of modeling errors, it is advisable to consider anisotropic conductivity for muscle tissue in subsequent simulations. To realize anisotropic muscle conductivity, we applied a novel approach based on flow simulations to approximate the muscle fiber orientation. This method can also be implemented in other models when no DTI data are available. Our study also highlights the necessity to achieve a more precise characterization of the anisotropic conductivity for the muscles in the neck.

We concluded that high-resolution individual models taking into account anisotropic volume conduction are suitable for research purposes in electrical ventilation. In clinical applications, however, it might not be possible to construct such individual models for practical reasons.

Furthermore, given the considerable influence of muscle anisotropy on phrenic nerve activation thresholds and potential co-activation, we conclude that the use of individual anatomical models is advisable in research on electric ventilation. For practical application, it is advisable to avoid electrode positioning directly above the muscle. Further stimulation protocol optimization may reduce co-activation. These insights bridge the gap between computational modeling and practical application, supporting the clinical stimulation protocol design.

## Supplementary Information

Below is the link to the electronic supplementary material.

Supplementary file 1.

## Data Availability

The data used in this publication are available upon request to the corresponding author.
