# Supplementary material for: Muscle anisotropy influences the phrenic nerve activation threshold in non-invasive electrical stimulation
Source: Med Biol Eng Comput. 2026 May 20;64(6):2377–91. doi: 10.1007/s11517-026-03584-2 (PMC13269526; doi:10.1007/s11517-026-03584-2)
Supplement: Supplementary file 1 [file 11517_2026_3584_MOESM1_ESM.pdf]

Supplementary Material

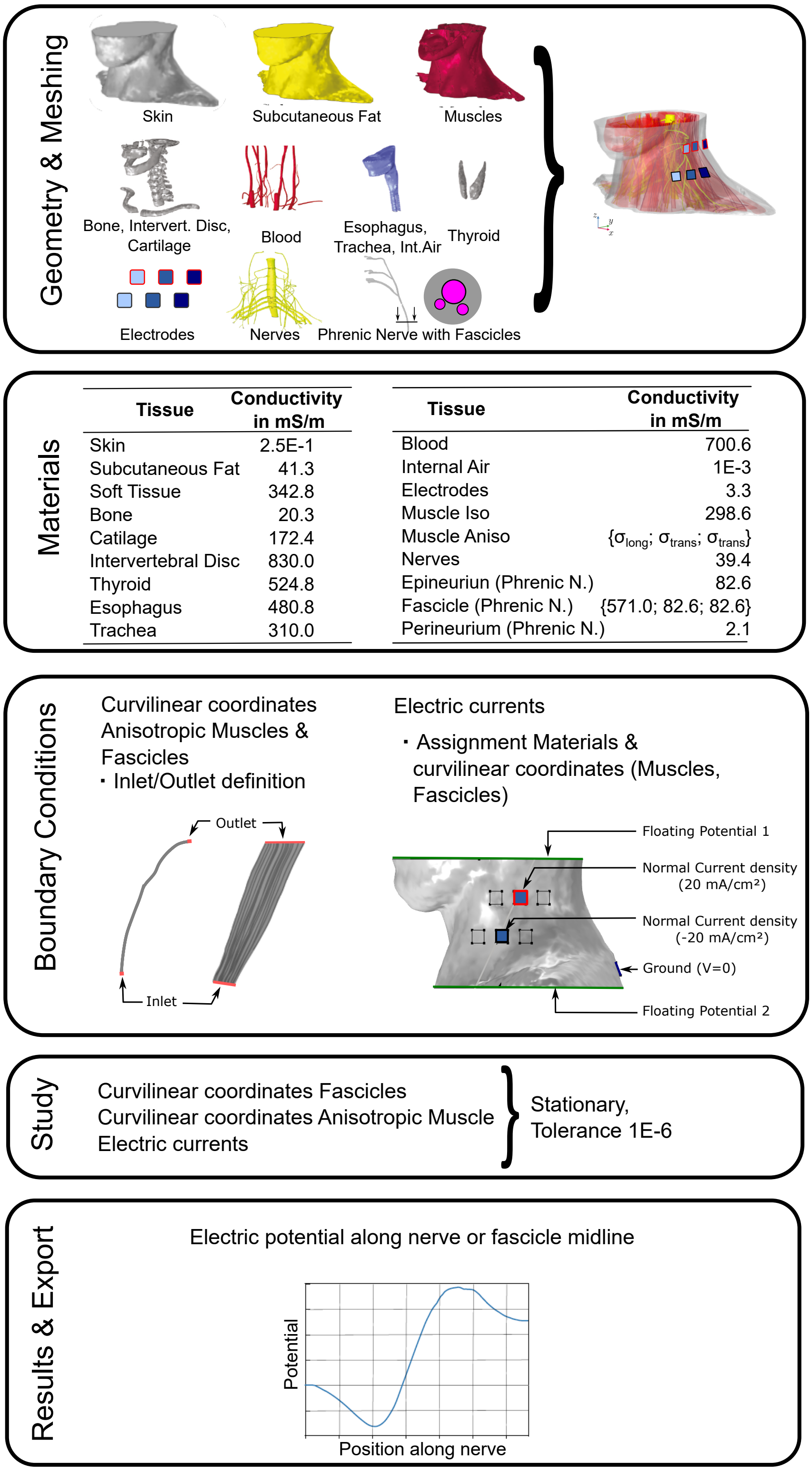

Figure A.1: Overview of the modeling steps for the FEM neck model. In the geometry step, the model is built based on tissue compartments from *BodyParts3D* and includes the fascicles within the phrenic nerve and the stimulation electrodes. An electric conductivity value is assigned to each compartment. The boundary conditions include creating curvilinear coordinates based on a flow simulation and an electric current interface to calculate the resulting potential distribution from the stimulation current. The study is calculated using a stationary solver and enhanced tolerance. The result is an electric potential calculated within the neck model and exported along specific coordinates on the midline of each nerve.

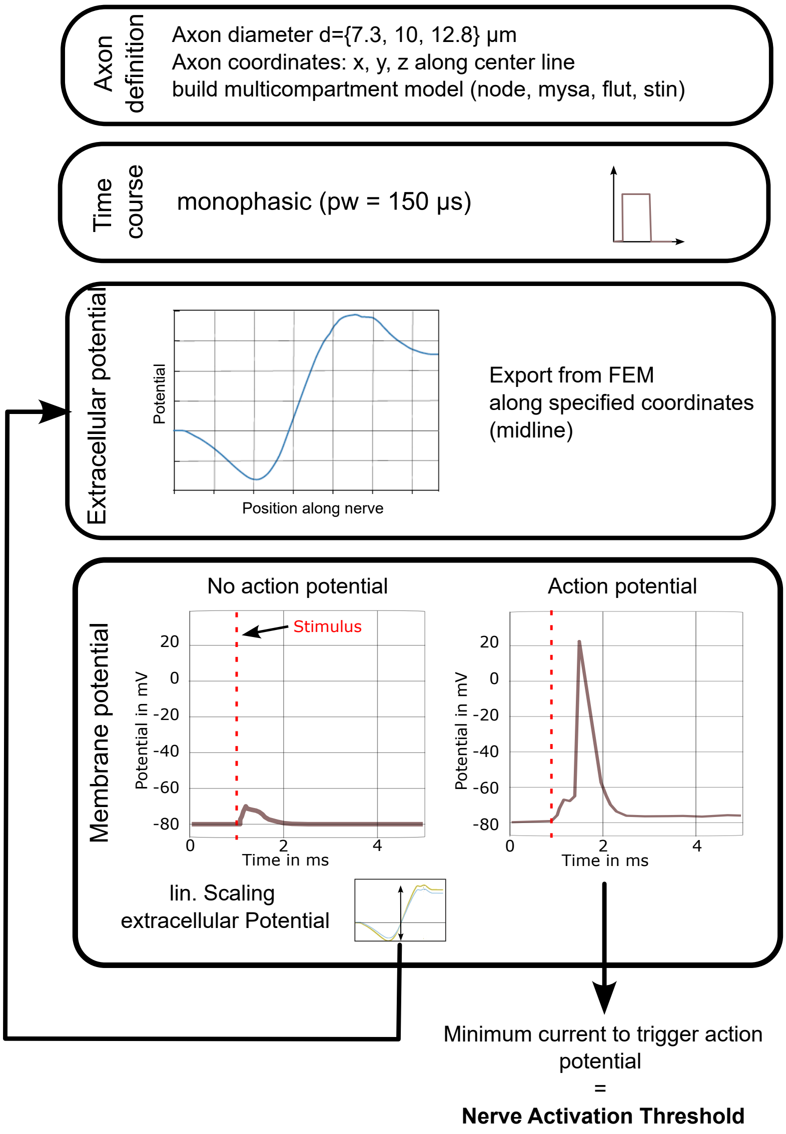

Figure A.2: Overview of the calculation of the nerve activation threshold. Axons are built based on the multi-compartment MRG model. The axon is defined based on fiber diameter and its three-dimensional course. The time course of the stimulation signal is defined. The extracellular potential (exported from the FEM model) is applied to each axon compartment, and the resulting membrane potential is evaluated. If no action potential is detected, the extracellular potential is increased iteratively until an action potential is triggered. The minimum stimulation current required to trigger an action potential is defined as the nerve activation threshold.

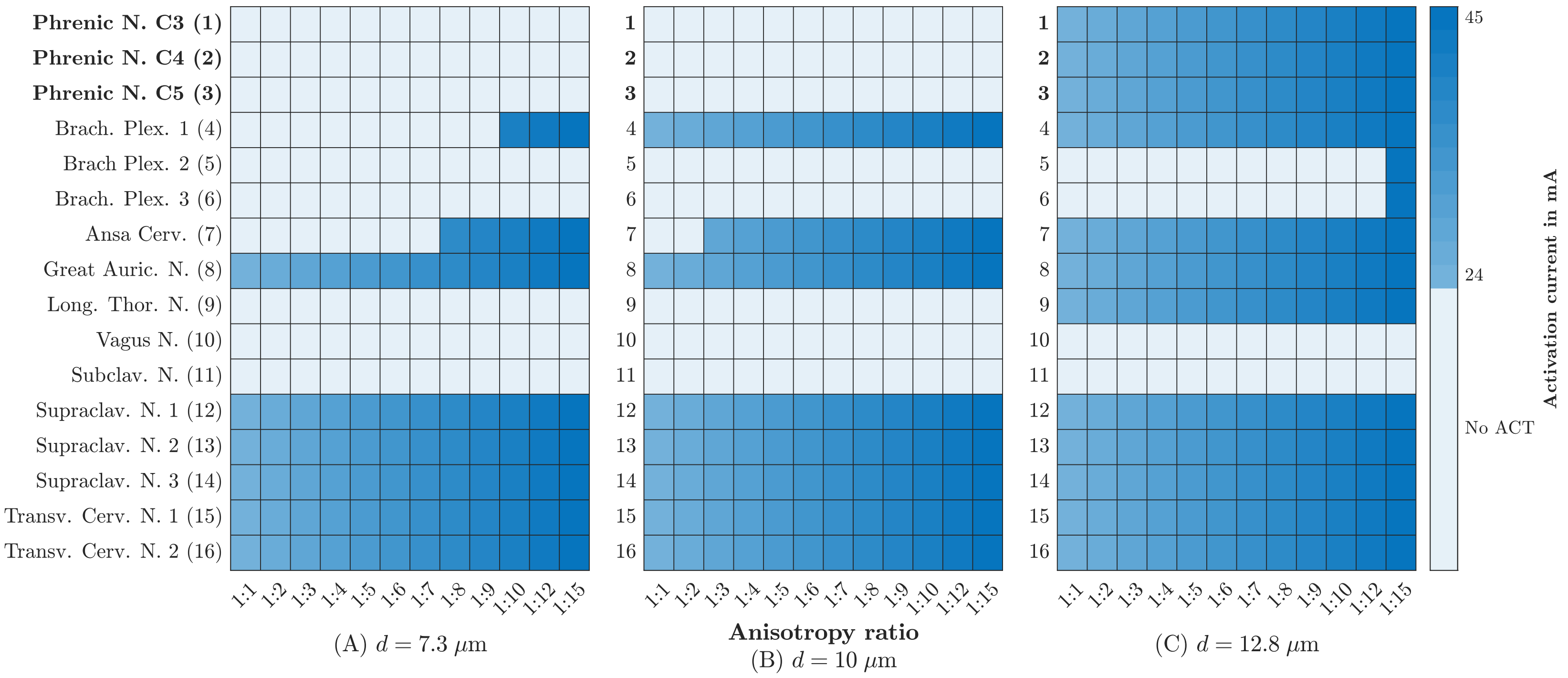

Figure A.3: Activated nerve segments when stimulation current was applied to activate phrenic nerve fibers with a diameter of 12.8 µm (activation current from 24 mA to 45 mA). The subplots summarize the activated and non-activated (No ACT) nerve segments at varying anisotropy ratios and three representative fiber diameters: 7.3 µm (A), 10 µm (B), and 12.8 µm (C). The target phrenic nerve fibers are shown in rows 1-3, and the off-target nerve segments are shown in rows 4-16. Nerve segments with smaller fiber diameters show less activation than those with larger diameters. Additionally, a higher anisotropy ratio leads to the activation of more off-target fibers (e.g., brachial plexus and ansa cervicalis) and consequently reduced selectivity.

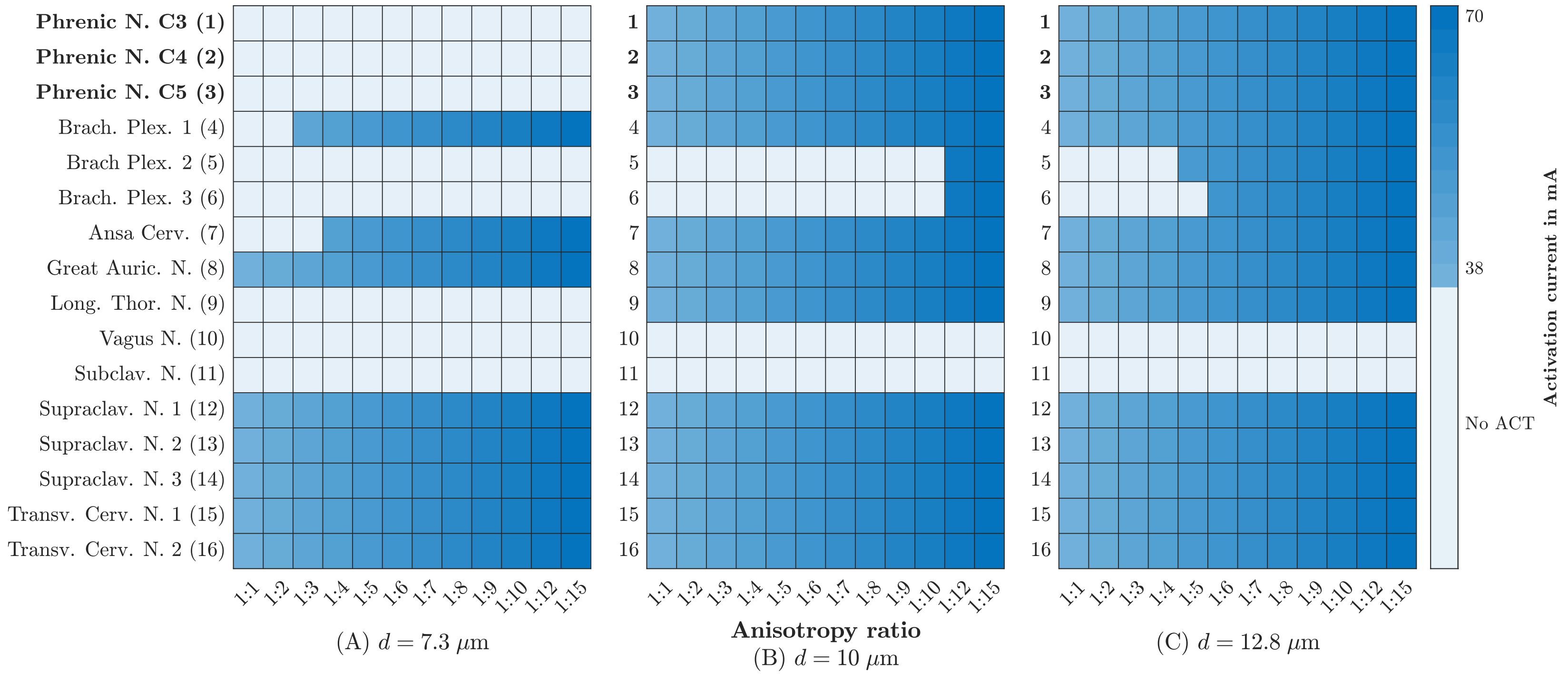

Figure A.4: Activated nerve segments when stimulation current was applied to activate phrenic nerve fibers with a diameter of 10 µm (activation current from 38 mA to 70 mA). The subplots summarize the activated and non-activated (No ACT) nerve segments at varying anisotropy ratios and three representative fiber diameters: 7.3 µm (A), 10 µm (B), and 12.8 µm (C). The target phrenic nerve fibers are shown in rows 1-3, and the off-target nerve segments are shown in rows 4-16. Nerve segments with smaller fiber diameters show less activation than those with larger diameters. Additionally, a higher anisotropy ratio leads to the activation of more off-target fibers (e.g., brachial plexus and ansa cervicalis) and consequently reduced selectivity.

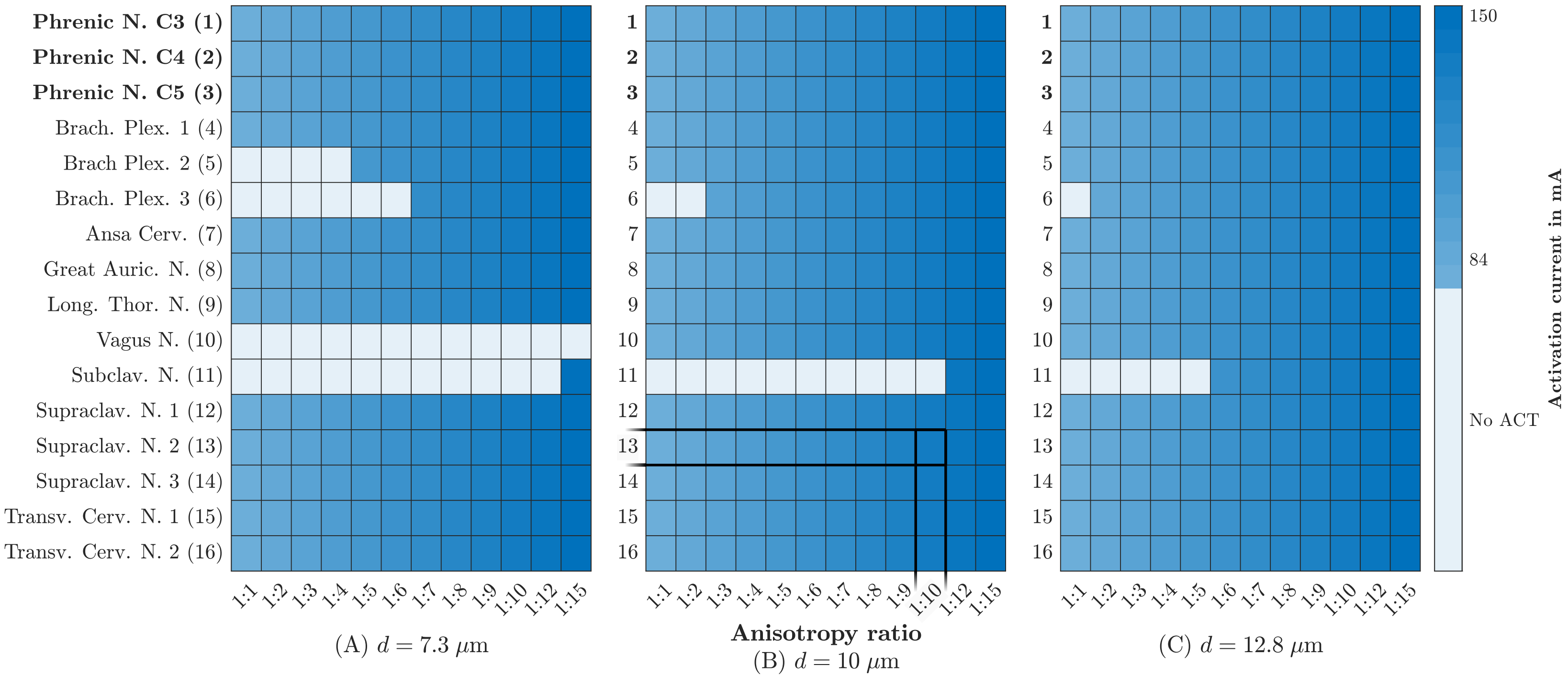

Figure A.5: Activated nerve segments when stimulation current was applied to activate phrenic nerve fibers with a diameter of 7.3 µm (activation current from 84 mA to 150 mA). The subplots summarize the activated and non-activated (No ACT) nerve segments at varying anisotropy ratios and three representative fiber diameters: 7.3 µm (A), 10 µm (B), and 12.8 µm (C). The target phrenic nerve fibers are shown in rows 1-3, and the off-target nerve segments are shown in rows 4-16. Nerve segments with smaller fiber diameters show less activation than those with larger diameters. Additionally, a higher anisotropy ratio leads to the activation of more off-target fibers (e.g., brachial plexus and subclavian nerve) and consequently reduced selectivity.
